# Sleep Apnea, Sleep Duration and Brain MRI Markers of Cerebral Vascular Disease and Alzheimer’s Disease: The Atherosclerosis Risk in Communities Study (ARIC)

**DOI:** 10.1371/journal.pone.0158758

**Published:** 2016-07-14

**Authors:** Pamela L. Lutsey, Faye L. Norby, Rebecca F. Gottesman, Thomas Mosley, Richard F. MacLehose, Naresh M. Punjabi, Eyal Shahar, Clifford R. Jack, Alvaro Alonso

**Affiliations:** 1 University of Minnesota, Minneapolis, MN, United States of America; 2 Johns Hopkins University, Baltimore, MD, United States of America; 3 University of Mississippi Medical Center, Jackson, MS, United States of America; 4 University of Arizona, Tucson, AZ, United States of America; 5 Mayo Clinic, Rochester, MN, United States of America; 6 Emory University, Atlanta, GA, United States of America; The Chinese University of Hong Kong, HONG KONG

## Abstract

**Background:**

A growing body of literature has suggested that obstructive sleep apnea (OSA) and habitual short sleep duration are linked to poor cognitive function. Neuroimaging studies may provide insight into this relation.

**Objective:**

We tested the hypotheses that OSA and habitual short sleep duration, measured at ages 54–73 years, would be associated with adverse brain morphology at ages 67–89 years.

**Methods:**

Included in this analysis are 312 ARIC study participants who underwent in-home overnight polysomnography in 1996–1998 and brain MRI scans about 15 years later (2012–2013). Sleep apnea was quantified by the apnea-hypopnea index and categorized as moderate/severe (≥15.0 events/hour), mild (5.0–14.9 events/hour), or normal (<5.0 events/hour). Habitual sleep duration was categorized, in hours, as <7, 7 to <8, ≥8. MRI outcomes included number of infarcts (total, subcortical, and cortical) and white matter hyperintensity (WMH) and Alzheimer’s disease signature region volumes. Multivariable adjusted logistic and linear regression models were used. All models incorporated inverse probability weighting, to adjust for potential selection bias.

**Results:**

At the time of the sleep study participants were 61.7 (SD: 5.0) years old and 54% female; 19% had moderate/severe sleep apnea. MRI imaging took place 14.8 (SD: 1.0) years later, when participants were 76.5 (SD: 5.2) years old. In multivariable models which accounted for body mass index, neither OSA nor abnormal sleep duration were statistically significantly associated with odds of cerebral infarcts, WMH brain volumes or regional brain volumes.

**Conclusions:**

In this community-based sample, mid-life OSA and habitually short sleep duration were not associated with later-life cerebral markers of vascular dementia and Alzheimer’s disease. However, selection bias may have influenced our results and the modest sample size led to relatively imprecise associations.

## Introduction

Since the 1960’s there has been a dramatic increase in the number of elderly individuals in the U.S. and worldwide, and this trend is projected to continue.[1, 2] The aging of the population is contributing to increasing numbers of people affected by dementia and mild cognitive impairment.[3, 4] Yet despite the immense and growing burden, gaps exist in our understanding of characteristics that lead to cognitive decline.

Accruing evidence has suggested a possible relation between abnormal sleep characteristics and cognitive impairment due to both cerebral vascular etiologies and Alzheimer’s disease. There are several mechanisms through which disordered sleep may lead to mild cognitive impairment and dementia.[5, 6] These include chronic nocturnal hypoxemia,[7] [8, 9] abnormal cerebral hemodynamic resulting from OSA,[10, 11] sleep fragmentation,[12] mediation through cardiovascular disease risk factors (e.g. hypertension, diabetes, inflammation), stroke (both clinical and subclinical),[9, 13–15] Aβ plaque build-up,[16] and interaction with the APOE ɛ4 risk allele.[17, 18] Neuroimaging studies may provide further insight into putative intermediate mechanisms. Existing human neuroimaging studies have suggested that OSA is associated with adverse brain morphology.[19–27] Unfortunately, these studies are limited in that they often use selected samples (e.g. from a sleep clinic), frequently have a limited number of participants (n generally <50) and were generally not prospective thus it is unclear whether the sleep characteristics preceded brain morphologic state.

Using data from the community-based ARIC study we explored the relation of OSA and abnormal (short or long) sleep duration with cerebral markers of cerebrovascular disease and Alzheimer’s disease measured via MRI as part of the ARIC neurocognitive study exam. Specifically, we tested the hypotheses that OSA and sleep duration measured at ages 54–73 years would be associated with a higher number of infarcts (total, subcortical, and cortical) and with greater white matter hyperintensity (WMH) and Alzheimer’s Disease signature region volumes measured about 15 years later (at ages 67–89 years).

## Methods

### Study Design

ARIC is a prospective epidemiological cohort which in 1987–1989 recruited 15,792 individuals from 4 U.S. communities.[28] Shortly after ARIC’s fourth clinical visit, which took place in 1996–1998, a total of 1,892 participants from the suburban Minneapolis, Minnesota and Washington County, Maryland field centers had in-home overnight polysomnography (PSG) as part of the Sleep Heart Health Study (SHHS).[29] Of those who took part in the sleep exam, a total of 1,723 had measures for OSA that met the quality control criteria.

In 2011–2013 all surviving ARIC participants were invited to take part in the ARIC Neurocognitive Study/ARIC Visit 5. A subset of Neurocognitive Study participants without contraindications were selected for brain MRI.[15] The subset included all those with low cognitive test scores, those with declines on longitudinally administered tests, those with a previous ARIC research brain MRI, and an age-stratified random sample of the remaining individuals. Sampling fractions for the random sample were set for participants <80 and ≥80 years of age to approximate the age distribution of those selected from the cognitively suspect group. They were modified slightly over the course of the study to achieve MRI scan completion goals. As detailed in the statistical analysis section, all analyses used inverse probability weighting (IPW) [30, 31] to account for attrition during follow-up, participation in the neurocognitive exam, and likelihood of selection to have a brain MRI. As such, the final estimates can be interpreted as being representative of all participants who had sleep study measures (n = 1723).

Of the 1,723 sleep study participants with OSA data, a total of 1,113 participated in the neurocognitive exam, and of these 317 were also selected for brain MRI. We further excluded one individual due to prevalent stroke at the time of the sleep study, two with missing brain volume measurements, and two who were lacking complete covariate information. Thus, 312 participants were included in our final analytic sample.

The University of Minnesota Institutional Review Board approved the present ARIC study, and all participants enrolled in the ARIC study have given their written informed consent.

### Sleep Measurements

The overnight unattended PSG was conducted using a portable monitor (PS-2 System; Compumedics Limited, Abbotsford, Victoria, Australia), using methods previously described.[32] As in prior analyses of SHHS data, an apnea was considered present if there was an absence or near absence of airflow (at least <25% of baseline) for ≥10 seconds.[29, 32] Hypopnea was defined as a decrease in the amplitude of the airflow below 70% of baseline for ≥10 seconds. The apnea-hypopnea index (AHI) was defined as the average number of obstructive apneas (any apnea, regardless of the oxygen desaturation level) plus hypopneas (with at least a 4% decrease in oxygen saturation), per hour of sleep. Participants were categorized into three OSA severity groups according to the AHI: <5.0 events/hr (normal), 5.0–14.9 events/hr (mild sleep apnea), ≥ 15.0 events/hr (moderate/severe sleep apnea). In sensitivity analyses we also separated moderate (15.0–29.9 events/hr) and severe (≥30.0 events/hr) sleep apnea. Central sleep apnea events, which were defined by the absence of airflow with no associated respiratory effort detected, were excluded.

Habitual sleep duration during the workdays and weekends was derived from the following questions on the SHHS Sleep Habits Questionnaire: *How much sleep do you usually get at night (or in your main sleep period)*: *on weekdays or workdays?* and *on weekends or nonwork days?* We calculated average usual sleep time per night (h) with the following formula: [(habitual total sleep time during the workdays)*5 + (habitual total sleep time during the weekends)*2]/7.

### Outcomes

Details of the ARIC brain imaging protocol, and of the definition and quantification of brain infarcts have been described previously.[15] In brief, MRI scans were performed in 2011–2013 as part of the neurocognitive exam at each site on 3 Tesla Siemens scanners (various models) using a common set of sequences that included 3-dimensional volumetric magnetization prepared gradient echo and fluid-attenuated inversion recovery sequences.

WMH burden was measured quantitatively using an algorithm developed at Mayo Clinic, Rochester,[33, 34] and WMH were defined as has been codified in recent guidelines.[35] All analyses involving WMH include total intracranial volume as a covariate. Brain infarcts were identified, counted, and measured by a trained imaging technician and confirmed by radiologists, as previously described.[36]

Freesurfer (version 5.1)[37] was used to calculate regional cortical volumes. There were 3 prespecified regions of interest: (1) the combined right and left hippocampal formations; (2) posterior region–inclusive of regions that are part of the posterior default mode network and are associated with Alzheimer disease from both right and left hemispheres: hippocampus, parahippocampal gyrus, entorhinal cortex, inferior parietal lobule, precuneus and cuneus; and (3) frontal region—including regions in the frontal lobe from both right and left hemispheres: rostral/caudal anterior cingulate, rostral/caudal midfrontal, lateral orbital frontal, medial orbital frontal, paracentral, pars opercularis, pars triangularis, precentral, superior frontal, and frontal pole. All volumes are expressed in cubic centimeters, and all models adjusted for total intracranial volume to account for differences in head size across participants.

### Confounders and effect modifiers

Covariate information was collected at ARIC visit 4, which took place shortly before the sleep study, unless otherwise noted. Information on age, sex, field center, and educational attainment (visit 1), physical activity (visit 3), ethanol intake, and smoking status were collected by questionnaire. BMI was calculated as weight (kg) over height^2^ (m). Diabetes was defined by fasting glucose ≥126 mg/dl, non-fasting glucose ≥200 mg/dL, self-reported physician diagnosis, or current use of medications for diabetes. Hypertension was defined by measured blood pressure >140/90 mmHg or use of anti-hypertensive medications. High-sensitivity C-reactive protein was measured using a nephelometric method on the Siemens Dade Behring BN II analyzer (Siemens Healthcare Diagnostics, Deerfield, IL). Prevalent CHD was defined by self-reported prior physician diagnosis of MI or coronary revascularization, prevalent MI by 12 lead ECG at visit 1, or an incident adjudicated CHD event between ARIC visits 1 and 4. Details of the measurement and classification of the APOE ɛ4 risk allele have been described previously.[38]

### Data analysis

Participant characteristics (% or mean ± SD) are provided stratified by OSA severity and, separately, by follow-up neurocognitive exam participation status. To evaluate the relation of abnormal sleep characteristics with cerebral markers of cerebrovascular disease and Alzheimer’s disease, logistic regression was used for dichotomous outcomes (i.e. any infarct, subclinical infarcts, large cortical infarcts, small cortical infarcts, or microhemorrhages) and linear regression was used for brain volume measurements. OSA and sleep duration were modeled categorically; indicator variables were included in the regression model.

If participants with brain MRI data are different from those without brain MRI data, selection bias may be present (see Figure 1 in reference by Wueave et al.[30]). In order to account for this potential selection bias, all primary analyses used IPW [30, 31] to adjust for a) selection for brain MRI and b) attrition due to either death or failure to attend the follow-up neurocognitive exam (censoring). Weights for each individual were the inverse of the product of the estimated probabilities of i) being alive at the time of the follow-up neurocognitive exam, ii) attending the follow-up neurocognitive examination (conditional on being alive at the time of the neurocognitive exam), and iii) being selected for the brain MRI (conditional on being alive at the time of the neurocognitive exam and participating in the neurocognitive examination). Characteristics included in the IPW models are provided in **S1 Methods.**

#### Analysis

A series of nested models were estimated. Model 1 adjusted for age, sex, field center, and educational attainment. Model 2 further adjusted for ethanol intake, smoking status, leisure time physical activity, and APOE ɛ4 risk allele. Model 3 additionally adjusted for body mass index. Model 4 also adjusted for characteristics believed to be on the causal pathway between OSA and cognitive decline (i.e. high-sensitivity C-reactive protein (CRP), diabetes mellitus, hypertension, and prevalent coronary heart disease).

## Results

**Table 1** presents baseline characteristics of sleep study participants (n = 1,723) categorized by whether they were included in the present analysis (18%; n = 312), attended the neurocognitive exam but did not have adequate information to be included in this analysis of brain MRI outcomes (46%; n = 801) or did not attend the neurocognitive study (35%; n = 610). Of those who did not attend the neurocognitive study, 63% had died. Relative to participants who attended the follow-up neurocognitive examination, those who did not tended to be, at the time of the sleep study, older, male, current smokers, more overweight, and have a worse overall cardiovascular risk factor profile. In spite of the sampling approach for selection into the brain MRI sample, participant characteristics at the time of the sleep study were similar for those who had brain MRI’s versus those who did not. Similarly, in terms of baseline cognitive test scores, among those who attended the neurocognitive exam, scores were similar between those who were selected for the brain MRI and those who were not (p all >0.05). However, participants who did not attend the neurocognitive exam scored more poorly on baseline cognitive tests than those who attended the neurocognitive exam (p all <0.05).

**Table 1:** Characteristics of the 1,723 participants at the time of the sleep exam (visit 4; 1996–1998), and over follow-up (1996–2012) stratified by neurocognitive follow-up exam (visit 5; 2011–2013) participation status.

	Neurocognitive exam and brain MRI (n = 312)	Neurocognitive exam but no brain MRI* (n = 801)	Did not participate in neurocognitive exam thus no Brain MRI (n = 610)
**Sleep Exam (1996–1998)**			
Age, years	61.7 ± 5.0	61.3 ± 5.1	65.3 ± 5.4
Female	53.6	54.4	47.9
Education < high school graduate	8.5	8.3	16.1
Leisure time physical activity	2.6 ± 0.8	2.6 ± 0.8	2.5 ± 0.8
Smoking status			
Current	7.6	8.9	13.8
Former	50.8	47.6	47.4
Never	41.6	43.5	38.9
Usual ethanol intake, g/week	43.1 ± 72.5	37.6 ± 70.3	37.7 ± 79.6
BMI, kg/m^2^	28.6 ± 5.0	28.5 ± 4.8	29.1 ± 5.4
Hypertension	32.5	35.3	48.5
Diabetes mellitus	8.8	9.1	18.0
Prior heart failure	0	0.8	2.6
Prior coronary heart disease	4.4	6.7	13.4
C-reactive protein	3.6 ± 4.6	3.7 ± 5.6	4.6 ± 6.2
Cognitive test scores, mean (SD)			
Delayed Word Recall	7.0 (5.3)	6.9 (1.5)	6.5 (2.1)
Word Fluency	37.0 (11.1)	37.5 (10.9)	34.4 (11.4)
Digit Symbol Substitution	49.9 (10.1)	50.9 (10.2)	44.8 (11.0)
APOE ɛ4 risk allele			
0	74.8	73.9	71.0
1	22.4	24.5	24.9
2	2.8	1.6	4.1
Prevalent stroke, n (%)	0	8 (1.0)	19 (3.2)
**Over Follow-Up (1996–2012)**			
Incident stroke, n (%)	10 (3.2)	27 (3.4)	62 (10.2)
Person-years	4858	12038	6453
Incidence rate (95% CI), per 1,000 p-y	2.1 (1.0–3.8)	2.2 (1.5–3.3)	9.6 (7.4–12.3)
Incident HF, n (%)	13 (4.1)	55 (6.9)	124 (20.3)
Incident CHD, n (%)	10 (3.2)	48 (6.0)	69 (11.3)
Dead by 2012, n (%)	0	16 (2.0)	384 (63.0)

Values correspond to mean ± SD or %

*Also included in this group are 5 individuals who had brain MRI but were not included in the primary analysis due to prevalent stroke at the time of the sleep study (n = 1), missing brain volume measurements (n = 2), or lacking complete covariate information (n = 2).

The 312 participants making up our final analytic sample were, at baseline, 61.7 (SD: 5.0) years old, and 54% female. A total of 60 (19%) had moderate/severe OSA (AHI ≥15), 88 (28%) had mild OSA (AHI ≥5–15), and 164 (53%) had no evidence of OSA (AHI <5). Relative to participants with no evidence of sleep disordered breathing, those with moderate/severe OSA tended to be male, had higher BMI’s and were more likely to be hypertensive **(Table 2)**. Baseline scores on the Digit Symbol Substitution tests were slightly better in those with a normal sleep breathing pattern as compared to those with mild or moderate/severe sleep apnea (p all <0.05). Baseline scores on the Delayed Word Recall and Word Fluency tests did not vary by sleep apnea category. By the time of the MRI exam, participants were on average 76.5 (SD: 5.2) years old.

**Table 2:** Demographic and clinical characteristics of the final analytic sample (n = 312) at the sleep exam (visit 4; 1996–1998), at the neurocognitive exam (2011–2013) and over follow-up (1996–2012), stratified by sleep exam obstructive sleep apnea categories.

	Normal	Mild	Moderate / Severe
	AHI <5	AHI 5 to <15	AHI ≥15
	(n = 164)	(n = 88)	(n = 60)
**Sleep Exam (1996–1998)**			
Age, years	61.1 ± 5.2	62.4 ± 4.9	62.5 ± 4.5
Female	70.1	37.5	31.7
Education ≥ high school graduate	92.7	89.8	91.7
Leisure time physical activity	2.7 ± 0.8	2.5 ± 0.8	2.6 ± 0.8
Smoking status			
Current	11.6	1.1	6.7
Former	43.9	61.4	55.0
Never	44.5	37.5	38.3
Usual ethanol intake, g/week	42.3 ± 73.2	47.6 ± 79.1	40.2 ± 62.0
BMI, kg/m^2^	27.0 ± 4.7	29.3 ± 4.2	31.7 ± 5.3
Hypertension	29.9	37.5	33.3
Diabetes mellitus	7.3	11.4	8.3
Prior heart failure	0	0	0
Prior coronary heart disease	4.3	3.4	5.0
C-reactive protein	3.9 ± 5.5	3.0 ± 2.9	4.0 ± 4.0
Cognitive test scores, mean (SD)			
Delayed Word Recall	7.4 (7.2)	6.6 (1.4)	6.6 (1.4)
Word Fluency	37.6 (11.9)	36.6 (11.1)	36.4 (8.8)
Digit Symbol Substitution	51.4 (10.2)	48.2 (10.8)	48.4 (8.1)
APOE ɛ4 risk allele			
0	70.7	79.6	76.7
1	25.0	18.2	23.3
2	4.3	2.3	0
**Neuroimaging (2011–2013)**			
Any infarction	23.8	27.3	16.7
Subcortical Infarction	18.9	18.2	10.0
Large cortical Infarction	2.4	4.6	3.3
Small cortical infarction	5.5	8.0	8.3
Microhemorrhage	24.4	27.3	18.3
White matter hyperintensity volume, cm^3^	16.7 ± 16.2	19.1 ± 19.5	15.5 ± 17.8
Estimated intracranial volume in cm^3^	1397.3 ± 156.9	1454.1 ± 156.2	1460.4 ± 132.8
Total brain volume in cm^3^	1024.6 ± 105.9	1049.3 ± 114.3	1066.2 ± 100.6
Temporal lobe cortical volume in cm^3^	102.8 ± 11.1	105.6 ± 12.2	105.1 ± 11.0
Parietal lobe cortical volume in cm^3^	108.1 ± 11.8	110.3 ± 12.4	111.1 ± 11.4
Occipital lobe cortical volume in cm^3^	41.7 ± 5.3	41.9 ± 5.8	44.1 ± 5.4
Frontal lobe cortical volume in cm^3^	151.4 ± 15.2	155.2 ± 15.2	156.9 ± 13.9
Deep grey matter in cm^3^	42.9 ± 4.3	43.6 ± 4.1	43.7 ± 4.1
AD signature region volume in cm^3^	60.2 ± 6.8	61.2 ± 6.8	61.7 ± 6.4
Hippocampal volume in cm^3^	6.9 ± 1.0	6.9 ± 1.0	6.8 ± 0.9
**Over follow-up (1996–2012)**			
Incident stroke	3.7	4.6	0
Incident HF	3.7	4.6	5.0
Incident CHD	1.8	4.6	5.0

Values correspond to mean ± SD or %

**Table 3** presents odds ratios and 95% confidence intervals from the weighted logistic regression models for total, subcortical and cortical infarcts, stratified by OSA categories. Contrary to our initial hypothesis, OSA was associated with lower odds of infarcts, particularly subcortical infarcts. Associations of OSA severity with brain MRI volume measurements are shown in **Table 4** and **S1 Table.** Estimates and 95% confidence intervals in z-scores from inverse probability weighted linear regression models for Brain MRI volume measurement z-scores (2011–2013), stratified by OSA categories (1996–1998). OSA severity was not related to any of the brain volumes explored (i.e. total brain volume, deep gray matter, hippocampal volume, and cortical volumes of the temporal, parietal, occipital and frontal lobes.

**Table 3:** Odds ratio and 95% confidence interval from inverse probability weighted logistic regression models for Brain MRI measures (2011–2013), stratified by OSA categories (1996–1998).

	Normal	Mild	Moderate / Severe	
	AHI < 5	AHI 5 to <15	AHI ≥15	P for trend*
	(n = 164)	(n = 88)	(n = 60)	
Any infarct, N (%)	39 (23.7)	24 (22.7)	10 (16.7)	
Model 1	Reference	0.55 (0.25–1.19)	0.45 (0.16–1.25)	0.07
Model 2	Reference	0.51 (0.23–1.12)	0.38 (0.13–1.05)	0.05
Model 3	Reference	0.52 (0.24–1.17)	0.41 (0.13–1.25)	0.08
Model 4	Reference	0.52 (0.24–1.14)	0.44 (0.15–1.31)	0.10
Subcortical Infarct, N (%)	31 (18.9)	16 (18.2)	6 (10.0)	
Model 1	Reference	0.52 (0.23–1.18)	0.35 (0.10–1.27)	0.08
Model 2	Reference	0.47 (0.20–1.09)	0.28 (0.08–0.95)	0.03
Model 3	Reference	0.50 (0.22–1.14)	0.32 (0.09–1.15)	0.05
Model 4	Reference	0.51 (0.23–1.17)	0.33 (0.09–1.17)	0.05
Cortical Infarction (either small and/or large), N (%)	11 (6.7)	9 (10.2)	7 (11.7)	
Model 1	Reference	1.43 (0.47–4.33)	2.29 (0.62–8.38)	0.22
Model 2	Reference	1.35 (0.45–4.06)	2.14 (0.67–6.89)	0.21
Model 3	Reference	1.36 (0.44–4.19)	2.15 (0.57–8.06)	0.27
Model 4	Reference	1.25 (0.42–3.76)	2.42 (0.68–8.63)	0.21

*P for trend from logistic regression model with obstructive sleep apnea modeled as an ordinal variable

Model 1 adjusted for age, sex, field center, and educational ascertainment; Model 2 adjusted for Model 1 and ethanol intake, smoking status, leisure time physical activity, and APOE ɛ4 risk allele; Model 3 adjusted for Model 2 and body mass index; Model 4 adjusted for Model 3 and high-sensitivity C-reactive protein, diabetes mellitus, hypertension, and prevalent coronary heart disease.

**Table 4:** Estimates and 95% confidence intervals in z-scores from inverse probability weighted linear regression models for Brain MRI volume measurements (2011–2013), stratified by OSA categories (1996–1998).

	Normal	Mild	Moderate / Severe	
	AHI < 5	AHI 5 to <15	AHI ≥15	P for trend*
	(n = 164)	(n = 88)	(n = 60)	
White matter hyperintensity volume				
Model 1	Reference	-0.12 (-0.34 to 0.11)	-0.25 (-0.51 to 0.01)	0.06
Model 2	Reference	-0.13 (-0.35 to 0.10)	-0.28 (-0.52 to -0.03)	0.03
Model 3	Reference	-0.08 (-0.31 to 0.14)	-0.18 (-0.46 to 0.10)	0.21
Model 4	Reference	-0.07 (-0.29 to 0.15)	-0.18 (-0.45 to 0.10)	0.20
AD signature region (gray matter) volume				
Model 1	Reference	0.02 (-0.17 to 0.21)	0.12 (-0.09 to 0.34)	0.31
Model 2	Reference	-0.01 (-0.21 to 0.19)	0.11 (-0.10 to 0.33)	0.40
Model 3	Reference	-0.00 (-0.20 to 0.20)	0.13 (-0.11 to 0.37)	0.35
Model 4	Reference	-0.00 (-0.19 to 0.19)	0.12 (-0.12 to 0.36)	0.39

*P for trend from linear regression model with obstructive sleep apnea modeled as an ordinal variable

Model 1 adjusted for age, sex, field center, and educational ascertainment + TIV; Model 2 adjusted for Model 1 and ethanol intake, smoking status, leisure time physical activity, and APOE ɛ4 risk allele; Model 3 adjusted for Model 2 and body mass index; Model 4 adjusted for Model 3 and high-sensitivity C-reactive protein, diabetes mellitus, hypertension, and prevalent coronary heart disease.

For both categorical (Table 3) and continuous (Table 4 and **S1 Table.** Estimates and 95% confidence intervals in z-scores from inverse probability weighted linear regression models for Brain MRI volume measurement z-scores (2011–2013), stratified by OSA categories (1996–1998) brain outcomes, in sensitivity analyses we ran unweighted analyses, and explored alternate weighting options. Results were similar regardless of whether or how the weighting was specified. For instance, in unweighted analyses with model 3 adjustments and relative to those with a normal sleep breathing pattern, the OR for any infarct associated with moderate/severe OSA was 0.42 (0.17–1.02). We also conducted analyses separating moderate and severe OSA. However, as only 24 individuals had severe OSA precision was poor (data not shown); therefore we retained the combined moderate/severe OSA category for the primary analyses.

In this population means (SD) hours if sleep per night was 7.2 ± 1.0 hours per night. Of our sample 22.8% reported >7 hours/night, 40.8% 7 to <8 hours per night, and 36.5% ≥8 hours per night. Habitually short or long sleep duration was not related to either brain infarcts or brain volume measurements (**Tables 5** and **6**).

**Table 5:** Odds ratio and 95% confidence intervals from inverse probability weighted logistic regression models for Brain MRI measures (2011–2013), stratified by sleep duration categories (1996–1998).

	<7 hours	7 to <8 hours	≥8 hours	P for trend*
	(n = 71)	(n = 127)	(n = 114)	
Any infarct, N (%)	12 (16.9)	31 (24.4)	26 (22.8)	
Model 1	0.63 (0.25–1.57)	Reference	0.99 (0.48–2.05)	0.99
Model 2	0.65 (0.27–1.62)	Reference	0.99 (0.48–2.03)	0.99
Model 3	0.64 (0.26–1.56)	Reference	0.99 (0.48–2.04)	0.99
Model 4	0.69 (0.29–1.63)	Reference	0.97 (0.45–2.09)	0.92
Subcortical Infarct, N (%)	9 (12.7)	25 (19.7)	19 (16.7)	
Model 1	0.50 (0.17–1.47)	Reference	0.75 (0.34–1.65)	0.50
Model 2	0.59 (0.19–1.78)	Reference	0.79 (0.36–1.70)	0.56
Model 3	0.58 (0.20–1.69)	Reference	0.78 (0.36–1.69)	0.54
Model 4	0.64 (0.24–1.69)	Reference	0.73 (0.31–1.72)	0.47
Cortical Infarction (either small and/or large), N (%)	5 (7.0)	12 (9.5)	10 (8.8)	
Model 1	0.81 (0.23–2.90)	Reference	0.84 (0.26–2.66)	0.76
Model 2	0.82 (0.28–2.43)	Reference	0.79 (0.26–2.39)	0.68
Model 3	0.83 (0.28–2.48)	Reference	0.79 (0.26–2.38)	0.67
Model 4	0.75 (0.25–2.30)	Reference	0.78 (0.25–2.38)	0.66

*P for trend from linear regression model with obstructive sleep duration categories modeled as an ordinal variable

Model 1 adjusted for age, sex, field center, and educational ascertainment + TIV; Model 2 adjusted for Model 1 and ethanol intake, smoking status, leisure time physical activity, and APOE ɛ4 risk allele; Model 3 adjusted for Model 2 and body mass index; Model 4 adjusted for Model 3 and high-sensitivity C-reactive protein, diabetes mellitus, hypertension, and prevalent coronary heart disease.

**Table 6:** Estimates and 95% confidence intervals in z-scores from inverse probability weighted linear regression models for Brain MRI volume measurements (2011–2013), stratified by sleep duration categories (1996–1998).

	<7 hours (n = 71)	7 to <8 hours (n = 127)	≥8 hours (n = 114)	P for trend*
White matter hyperintensity volume				
Model 1	0.014 (-0.238 to 0.265)	Reference	-0.079 (-0.285 to 0.127)	0.45
Model 2	0.014 (-0.254 to 0.282)	Reference	-0.114 (-0.317 to 0.089)	0.27
Model 3	0.002 (-0.264 to 0.268)	Reference	-0.110 (-0.309 to 0.090)	0.28
Model 4	0.009 (-0.253 to 0.272)	Reference	-0.117 (-0.314 to 0.081)	0.24
AD signature region volume				
Model 1	-0.168 (-0.376 to 0.041)	Reference	-0.070 (-0.242 to 0.102)	0.44
Model 2	-0.192 (-0.404 to 0.021)	Reference	-0.108 (-0.276 to 0.061)	0.22
Model 3	-0.191 (-0.403 to 0.021)	Reference	-0.108 (-0.287 to 0.060)	0.22
Model 4	-0.180 (-0.390 to 0.030)	Reference	-0.119 (-0.291 to 0.055)	0.19

*P for trend from linear regression model with obstructive sleep duration categories modeled as an ordinal variable

Model 1 adjusted for age, sex, field center, and educational ascertainment + TIV; Model 2 adjusted for Model 1 and ethanol intake, smoking status, leisure time physical activity, and APOE ɛ4 risk allele; Model 3 adjusted for Model 2 and body mass index; Model 4 adjusted for Model 3 and high-sensitivity C-reactive protein, diabetes mellitus, hypertension, and prevalent coronary heart disease.

## Discussion

Intriguing prior evidence has suggested that abnormal indices of sleep quality and quantity may lead to adverse brain morphologic changes. However, in this community-based sample of 312 individuals there was no evidence that OSA or abnormal habitual sleep duration were associated with greater likelihood of cerebral markers of cerebrovascular disease or Alzheimer’s disease, which were measured 15 years later. We consider the 15-year follow-up an important strength of our study, since dementia and mild cognitive impairment are believed to have a long pre-clinical phase and the etiologically relevant stage of life may be middle-age, as has been suggested with other risk factors for cognitive decline (e.g. hypertension,[39–41] diabetes,[41–45] smoking[41, 46]).

Counter to our findings, several prior studies have supported the hypothesis that sleep disordered breathing is associated with adverse brain morphological changes. Small neuroimaging studies have reported that silent infarcts are more common among patients with obstructive sleep apnea (OSA) than among controls,[26, 27] and white matter disease severity is correlated with the number of apnea/hypopnea events in patients with prevalent stroke.[19] Numerous other small studies have reported OSA patients to have smaller gray-matter volumes/densities than controls in a variety of brain regions,[20–25] with the hippocampus most frequently noted. The consistency with which structural differences have been observed between those with OSA and controls is intriguing; however, existing studies are limited in that they are not prospective, often use selected populations (e.g. from a sleep clinic), and have small sample sizes (n generally <50). In concordance with the human literature, animal models have shown that both intermittent hypoxia[47, 48] and sleep fragmentation[49, 50] can independently lead to neuronal loss in the hippocampus and prefrontal cortex. However, similar to the findings presented herein, in a prior analysis of nearly 2,000 ARIC participants who had sleep studies, there was no association of OSA or sleep quantity and quality with 15-year change in cognitive test scores.[51]

When interpreting our results, issues of power and selection bias require careful consideration. Although our sample of over 300 individuals is large relative to most prior studies, only 24 participants had severe OSA. As such, this necessitated aggregating moderate and severe OSA, which may have masked associations only appearing at the severe end of the spectrum. Additionally, our modest sample size resulted in effect estimates that had relatively little precision. Selection bias may also have hampered our ability to detect an association between midlife sleep characteristics and late-life brain morphology, since of the original SHHS participants, one-third did not attend the follow-up neurocognitive exam due to either death or attrition. We attempted to account for selection bias by employing IPW models. A limitation of this approach is that including weights can may increase the variance.[52] Overall, however, results of the IPW analyses were similar to those from standard analyses. Other limitations include a single assessment of sleep and incomplete information on OSA treatment during the follow-up period.

Despite these limitations our data is unique in that a) the design was prospective, therefore unlike cross-sectional studies temporality is clear, with midlife sleep characteristics assessed about 15 years prior to brain MRI indices, b) the time-span from mid-life to late-life is hypothesized to be etiologically relevant in considerations of risk factors for cognitive decline and dementia, c) objective sleep measurements were conducted though in-home polysomnography, and d) state-of-the-art brain MRI assessment was used.

In conclusion, in this community-based sample we found no evidence that midlife OSA and short sleep duration increase risk of late-life brain morphologic indices of vascular dementia and Alzheimer’s disease.

## Supporting Information

S1 Methods (DOCX)

S1 Table (DOCX)
