# Supplementary material for: Sleep Apnea, Sleep Duration and Brain MRI Markers of Cerebral Vascular Disease and Alzheimer’s Disease: The Atherosclerosis Risk in Communities Study (ARIC)
Source: PLoS One. 2016 Jul 14;11(7):e0158758. doi: 10.1371/journal.pone.0158758 (PMC4944966; doi:10.1371/journal.pone.0158758)
Supplement: S1 Methods — (DOCX) [file pone.0158758.s001.docx]

**Supplemental Methods – Analysis**

Inverse Probability Weighting

Logistic models for visit 5 participation, conditional on survival to visit 5, and survival included the follow variables measured at or before visit 4: age, sex, center, high school graduate, APOE, CRP, smoking status, ethanol intake, BMI, diabetes, hypertension, stroke, heart failure, coronary heart disease, physical activity, HDL and LDL cholesterol, estimated glomerular filtration rate, OSA, global z-score for cognitive tests; variables from the annual follow up included: stroke, chronic lung disease, diabetes, hypertension, coronary heart disease, retirement status, health status, smoking status, cancer diagnosis, entered a nursing home, and number of hospitalizations after visit 4. For some of these variables, i.e., diabetes, a positive response at any time was considered evidence of diabetes. For the following variables, the most recent information was considered: retirement status, health status, smoking status, and marital status (married/single/widowed/divorced/separated).

The stabilized weights included age, sex, APOE and high school graduate.

The c-statistics for visit 5 participation and survival were 0.75 and 0.93, respectively.

**Weights mean (SD) by Visit 5 status**

Visit 5 attendee: 5.43(4.54); min = 2.01, max = 60.13

Visit 5 nonattendee: 2.55(5.13); min = 1.03, max = 121.6

**Stabilized weights mean (SD) by Visit 5 status**

Visit 5 attendee: 0.97(0.53); min = 0.32, max = 5.81

Visit 5 nonattendee: 1.20(1.61); min = 0.34, max = 37.74
