# Supplementary material for: Sleep Apnea, Sleep Duration and Brain MRI Markers of Cerebral Vascular Disease and Alzheimer’s Disease: The Atherosclerosis Risk in Communities Study (ARIC)
Source: PLoS One. 2016 Jul 14;11(7):e0158758. doi: 10.1371/journal.pone.0158758 (PMC4944966; doi:10.1371/journal.pone.0158758)
Supplement: S1 Table — (DOCX) [file pone.0158758.s002.docx]

**Supplemental Table 1**. Estimates and 95% confidence intervals in z-scores from inverse probability weighted linear regression models for Brain MRI volume measurement z-scores (2011-2013), stratified by OSA categories (1996-1998).

|  | Normal  AHI < 5  (n = 164) | Mild  AHI 5 to <15  (n = 88) | Moderate / Severe  AHI ≥15  (n=60) | P for trend* |
| --- | --- | --- | --- | --- |
|  |  |  |  |  |
|  |  |  |  |  |
| Total brain volume | |  |  |  |
| Model 1 | Reference | 0.033 (-0.103 to 0.170) | 0.116 (-0.025 to 0.256) | 0.13 |
| Model 2 | Reference | 0.025 (-0.113 to 0.163) | 0.123 (-0.016 to 0.262) | 0.11 |
| Model 3 | Reference | 0.019 (-0.122 to 0.161) | 0.111 (-0.069 to 0.290) | 0.27 |
| Model 4 | Reference | 0.021 (-0.118 to 0.161) | 0.101 (-0.081 to 0.283) | 0.31 |
|  |  |  |  |  |
| Temporal lobe cortical volume | |  |  |  |
| Model 1 | Reference | 0.101 (-0.064 to 0.265) | 0.008 (-0.209 to 0.224) | 0.72 |
| Model 2 | Reference | 0.071 (-0.098 to 0.240) | 0.009 (-0.197 to 0.215) | 0.78 |
| Model 3 | Reference | 0.094 (-0.078 to 0.266) | 0.061 (-0.165 to 0.287) | 0.47 |
| Model 4 | Reference | 0.088 (-0.079 to 0.256) | 0.058 (-0.168 to 0.284) | 0.49 |
|  |  |  |  |  |
| Parietal lobe cortical volume | |  |  |  |
| Model 1 | Reference | 0.033 (-0.168 to 0.234) | 0.143 (-0.133 to 0.420) | 0.33 |
| Model 2 | Reference | -0.001 (-0.210 to 0.208) | 0.132 (-0.136 to 0.340) | 0.40 |
| Model 3 | Reference | 0.015 (-0.196 to 0.223) | 0.169 (-0.128 to 0.465) | 0.32 |
| Model 4 | Reference | -0.007 (-0.191 to 0.178) | 0.154 (-0.131 to 0.438) | 0.35 |
|  |  |  |  |  |
| Occipital lobe cortical volume | |  |  |  |
| Model 1 | Reference | -0.140 (-0.395 to 0.115) | 0.174 (-0.029 to 0.376) | 0.33 |
| Model 2 | Reference | -0.164 (-0.414 to 0.085) | 0.155 (-0.053 to 0.363) | 0.43 |
| Model 3 | Reference | -0.180 (-0.416 to 0.055) | 0.119 (-0.117 to 0.354) | 0.69 |
| Model 4 | Reference | -0.168 (-0.393 to 0.058) | 0.097 (-0.131 to 0.324) | 0.76 |
|  |  |  |  |  |
| Frontal lobe cortical volume | |  |  |  |
| Model 1 | Reference | 0.079 (-0.120 to 0.278) | 0.160 (-0.053 to 0.373) | 0.15 |
| Model 2 | Reference | 0.051 (-0.153 to 0.255) | 0.162 (-0.057 to 0.380) | 0.18 |
| Model 3 | Reference | 0.073 (-0.132 to 0.278) | 0.212 (-0.004 to 0.428) | 0.08 |
| Model 4 | Reference | 0.032 (-0.156 to 0.219) | 0.170 (-0.039 to 0.380) | 0.15 |
|  |  |  |  |  |
| Deep grey matter | |  |  |  |
| Model 1 | Reference | -0.117 (-0.284 to 0.051) | -0.073 (-0.266 to 0.120) | 0.31 |
| Model 2 | Reference | -0.133 (-0.294 to 0.029) | -0.078 (-0.266 to 0.110) | 0.26 |
| Model 3 | Reference | -0.138 (-0.305 to 0.028) | -0.091 (-0.300 to 0.119) | 0.26 |
| Model 4 | Reference | -0.174 (-0.345 to -0.004) | -0.113 (-0.324 to 0.099) | 0.17 |
|  |  |  |  |  |
| Hippocampal volume | |  |  |  |
| Model 1 | Reference | 0.188 (-0.033 to 0.409) | -0.053 (-0.311 to 0.205) | 0.43 |
| Model 2 | Reference | 0.131 (-0.101 to 0.358) | 0.042 (-0.226 to 0.311) | 0.58 |
| Model 3 | Reference | 0.148 (-0.084 to 0.380) | 0.080 (-0.219 to 0.380) | 0.45 |
| Model 4 | Reference | 0.104 (-0.127 to 0.334) | 0.029 (-0.253 to 0.312) | 0.70 |

*P for trend from linear regression model with obstructive sleep apnea modeled as an ordinal variable

Model 1 adjusted for age, sex, field center, and educational ascertainment + TIV

Model 2 adjusted for Model 1 and ethanol intake, smoking status, leisure time physical activity, and APOE ɛ4 risk allele

Model 3 adjusted for Model 2 and body mass index

Model 4 adjusted for Model 3 and high-sensitivity C-reactive protein, diabetes mellitus, hypertension, and prevalent coronary heart disease
